# An overview of mental health care system in Kilifi, Kenya: results from an initial assessment using the World Health Organization’s Assessment Instrument for Mental Health Systems

**DOI:** 10.1186/s13033-017-0135-5

**Published:** 2017-04-17

**Authors:** Mary A. Bitta, Symon M. Kariuki, Eddie Chengo, Charles R. J. C. Newton

**Affiliations:** 1 KEMRI-Wellcome Trust Research Programme, Centre for Geographic Medicine Research (Coast), P O Box 230, Kilifi, 80108 Kenya; 2 Ubuntu Afya Clinic for People with Epilepsy, Malindi, Kenya; 3 Department of Psychiatry, University of Oxford, Oxford, UK

**Keywords:** Kilifi, Kenya, Global mental health, Mental health systems, WHO-AIMS

## Abstract

**Background:**

Little is known about the state of mental health systems in Kenya. In 2010, Kenya promulgated a new constitution, which devolved national government and the national health system to 47 counties including Kilifi County. There is need to provide evidence from mental health systems research to identify priority areas in Kilifi’s mental health system for informing county health sector decision making. We conducted an initial assessment of state of mental health systems in Kilifi County and documented resources, policy and legislation and spectrum of mental, neurological and substance use disorders.

**Methods:**

This was a pilot study that used the brief version of the World Health Organization’s Assessment Instrument for Mental Health Systems Version 2.2 to collect data. Data collection was based on the year 2014.

**Results:**

Kilifi county has two public psychiatric outpatient units that are part of general hospitals. There is no standalone mental hospital in Kilifi. There are no inpatients or community based facilities for people with mental health problems. Although the psychiatric facilities in Kilifi have an essential drugs list, supply of drugs is erratic with frequent shortages. There is no psychiatrist or psychologist in Kilifi with only two psychiatric nurses for a population of approximately 1.2 million people. Schizophrenia was the commonest reason for visiting outpatient facilities (47.1%) while suicidal ideation was the least common (0.4%). Kenya’s mental health policy, which is being used by Kilifi County, is outdated and does not cater for the current mental health needs of Kilifi. There is no specific legislation to protect the rights of people with mental health problems. No budget exists specifically for mental health care. There have been no efforts to integrate mental health care into primary care in Kilifi, and there is no empirical research work to evaluate its feasibility.

**Conclusion:**

There is an urgent need to increase resources allocated for mental health in particular infrastructure and human resource. Policy and legislations need to be established to protect the rights of people with mental illnesses, and mental health should be integrated with primary care to increase access to services.

## Background

The 2001 World Health report by World Health Organization (WHO) [1] focussed on Mental Health and highlighted the dire state of mental health care globally and the need to direct more efforts towards improving mental health care. Ten key recommendations were made which culminated in the formation of the WHO Assessment Instrument for Mental Health Systems (WHO-AIMS) to assess the state of mental health services especially in resource poor settings. The WHO-AIMS [2] is a tool used to collect essential information about the mental health system of a country or region. The aim of collecting this information is to provide a basis for improving mental health systems and for monitoring change. A brief version of this instrument is recommended in four circumstances: (1) for research which is limited to a single part of the mental health system; (2) when carrying out an initial assessment with plans of following this up with a full version; (3) if mental health resources in that country or region are extremely limited; and (4) if the brief version is used together with another WHO-AIMS module.

Kenya is a country in East Africa which according to the World Bank is classified as a lower middle income country [3]. As of 2013, Kenya’s population was approximately 41.8 million (50% males) with 24% living in urban areas. The life expectancy is 62 years for males and 65 years for females. Protestants, Catholics, Muslims, Hindu, and Traditional Beliefs are the main forms of religion [4]. In 2010, a new constitution was enacted in Kenya, introducing a devolved system of governance, creating 47 administrative units called counties [5]. The health care system was thus devolved to the counties. A direct impact of devolution on health care was the inequitable distribution of resources, based on geographical boundaries.

Kenya, like many other low- and middle-income countries, has a very under-resourced mental health care system. There are 75 psychiatrists nation-wide and most of them reside in or near major cities. Mathari Mental Hospital, which is the only stand-alone public teaching and referral mental hospital in the country, is situated in Nairobi County 500 km from Kilifi county. Nairobi is also the only County with tertiary teaching facilities for training psychiatrists (the University of Nairobi). This leaves other Counties such as Kilifi lacking essential mental health services. Therefore, there is need to assess the prevailing situation for mental health care in poor counties such as Kilifi.

Our study aims to (1) understand the spectrum of mental disorders and the available resources, which will inform planning of mental health services; and (2) provide a basis for monitoring progress in mental health care services in light of a devolved health care system.

## Methods

### Study area and population

This study was conducted in Kilifi County, which is located on the Kenyan Coast. Kilifi covers an area of approximately 12,246 km^2^ and has a population of approximately 1.12 million people (2.9% of the national population), 48.3% being males [6]. The predominant inhabitants (approximately 80%) are from the Mijikenda community (mainly the Giriama, Chonyi and Kauma). There are also other groups of Swahili-Arab descendants, some Indians, Europeans, mainly British, German and Italian. The main economic activities are tourism, fishing and subsistence farming [7]. The prevalence of poverty (adult equivalent poverty head count) is 78.4% compared to the national proportion of 45.9%. It is ranked 8th poorest among 47 counties in Kenya. Climate conditions vary across the year and the region, but it is generally warm throughout the year [8]. Health care is structured into a County referral facility, a sub-County referral facility, primary care facilities and community units. The county executive committee is the governing authority. Health financing is mainly from national revenue although the County also receives grants and donations form charitable entities, raises money through taxes and licences and through loans. Additionally, the County is entitled to the Equalisation fund, which is a conditional grant given by the national government to marginalized areas. It aims to accelerate level of services to bring them up to par with the rest of the country.

### Study design

This was a quantitative study which used the brief version of the World Health Organization Assessment Instrument for Mental Health Systems (WHO-AIMS) Version 2.2 to collect data [2]. We also used brief unstructured interviews to clarify the quantitative data where it were inconsistent. The WHO-AIMS tool is designed to assess key components of a mental health system especially in resource poor settings. It comprises 6 domains, 28 facets and 156 items. The six domains are: (1) policy and legislative framework; (2) mental health services; (3) mental health in primary care; (4) human resources; (5) public education and links with other sectors; and (6) monitoring and research. The brief version contains 54 items that cover key areas in each domain.

We used the brief version because this was an initial assessment, which will be followed by a full version assessment and because the mental health resources in this region are extremely limited. Data were collected between September and November 2015 and were based on the year 2014. Data on the spectrum of psychiatric conditions were obtained both retrospectively (from records of admission in the previous year and) and prospectively (by systematically documenting admissions for at least 2-month period. Additionally, we summarised data on suicide from Kilifi’s Health and Demographic Surveillance System (KHDSS) because suicide has been linked to common mental health problems such as depression. The KHDSS has been described in detail elsewhere [9]. Information on the state of mental health was obtained from key informants from different departments within the county department of health.

Purposive sampling was used to identify four key informants, from the Ministry of Health of the County government of Kilifi, who provided all the information required to complete each of the 54 items. The four key informants were the Chief Officer of Health, a psychiatric nurse at the Kilifi County Psychiatric Outpatient Unit, a Clinical Officer managing a private epilepsy clinic in the county that also occasionally serves psychiatric patients and a representative from the finance department of the County government of Kilifi.

Four short questionnaires seeking specific information were generated from the 54 items in the brief version of the WHO-AIMS document, targeting each of the respondents. We also used data from Kilifi’s Health and Demographic Surveillance System.

### Data collection

The tools used to collect data were provided by the Department of Mental Health and Substance Abuse at WHO. Permission to conduct this situation analysis was sought from the Department of Health of the County government through the County Chief Officer of health. The Research Office of the KEMRI-Wellcome Trust Research Programme facilitated the initial engagements with the County Department of Health. Several preparatory meetings attended by representatives of Department of Health and the investigators were held to deliberate on the reasons and approaches for conducting the situation analysis on mental health. The first formal preparatory meeting was chaired by CN, and subsequent meetings by MB. To construct a database of admissions to the outpatient psychiatric clinics in Kilifi County, records were reviewed retrospectively by MB (guided by SK and CN) and in consultation with clinicians and nurses managing psychiatric clinics in the county. These records do not carefully document psychiatric diagnoses that prompted a prospective surveillance of admissions over a 2-month period (September–November 2015) to accurately monitor the types of diagnosis. Prospective surveillance of psychiatric conditions over the 2 months was done by MB who carefully documented the diagnosis of patients seen by the Psychiatric nurse at Kilifi County Hospital. The prospective surveillance generated frequency of psychiatric diagnoses at the outpatient facilities. The four Key informants were provided with instructions to independently to complete the short questionnaires. The first author (MB) completed the WHO-AIMS survey instrument using data from the self-administered questionnaires completed by the four key informants. Where the data from the questionnaires were unclear to provide information to some items in the WHO-AIMS, the first investigator (MB) undertook face-to-face interviews with the key informants, to obtain clarification. Where possible, data provided by key informants was cross validated against publicly available documents such as the county health budget. The survey, data collection and data analysis was led and undertaken by MB, CN and SK.

### Data analysis

Data were entered onto standardized WHO-AIMS 2.2 Excel spreadsheet. Descriptive statistical analyses were performed following aggregation of numerical data. Further analysis was done using Stata version 13 (Stata Corp, Texas, USA), to generate confidence intervals around the prevalence estimates of specific illnesses, based on the binominal distribution. The frequency of psychiatric admissions according to sex and being a child/adolescent was compared using a Pearson’s Chi square test or Fisher’s exact test, where the entries in a cell were infrequent (<5). A draft was prepared and discussed with the in-country-focal-point (the “in country focal point” is a well-connected senior and politically neutral person who leads the survey, and which is a requirement by the WHO-AIMS), the psychiatrist nurse and the county chief officer of health for comments. The final report on main findings conforms to the reporting guidelines of WHO.

## Results

### Policy and legislative framework

Kenya’s mental health policy, which is being applied in Kilifi County, was last revised in 1989. It includes the following components: (1) health promotion and illness prevention; (2) accessibility of care; (3) affordability and sustainability of care; (4) evidence based practise; and (5) stakeholder involvement both in the public and private sectors as well as families and consumers of mental health services. An essential medicines list exists with the following drugs being available: antipsychotics (haloperidol, chlorpromazine); anxiolytics (diazepam); anti-depressants (amitriptyline); mood stabilizers (carbamazepine, valproic acid); and antiepileptic drugs (phenobarbital, phenytoin, carbamazepine and valproic acid). Records for the drugs were not up-to-date and erratic supply of these drugs was documented.

No mental health plan exists and there is no disaster/emergency preparedness plan for mental health

### Mental health services

#### Organization of mental health services

The Division of Mental Health & Substance use Management Unit at the Ministry of Health is the mental health authority to which the county department of health reports. The Head of Mental Health & Substance use Management Unit heads the department. Mental health services are organized in terms of service areas namely Kilifi county and Malindi sub-county.

#### Mental health outpatient facilities

There are three outpatient mental health facilities available in the County; two facilities are run by the County government and there is one private facility which is run by a non-governmental organization. Additionally, there is an epilepsy research clinic run by the Neuroscience Department of the KEMRI-Wellcome Trust, which provides follow-up care for study participants with epilepsy. There is no facility allocated for children and adolescents. These facilities together treat about 180 psychiatric patients per 100,000 populations (95% CI 155–208), based on the number of outpatient visits only. Of all users treated in the mental health outpatient facilities during the 2 months’ prospective surveillance, 58.1% (95% CI 51.7–64.4%) are females and 41.9% (95% CI 35.6–48.3%) are males. There was no statistically significant difference in the frequency of mental disorders among males and females (p = 0.129), although the small sample size and convenience sampling prompts caution. Fifteen point eight percent (95% CI 11.5–21.0%) of all contacts are with patients 20 years or younger, 25.6% (95% CI 13.0–42.1%) of whom were children (<14 years).

Mental health problems treated in the health facilities were classified into either of the following priority psychiatric illnesses: depression, psychosis/schizophrenia, bipolar disorder, epilepsy, developmental disorders, behavioural/emotional disorders, dementia, alcohol and drug use disorders, self-harm/suicide, and other significant emotional or medically unexplained complaints. The users treated in outpatient facilities were commonly diagnosed with psychosis/schizophrenia and related disorders [47.1% (95% CI 40.7, 53.6)] and epilepsy [14.1% (95% CI 9.9, 19.1)] (Table 1).

**Table 1:** Spectrum of illnesses in an outpatient facility in Kilifi County over a 2-month period

Condition	Frequency (N = 242)	Prevalence (%)	95% confidence interval
Anxiety	1	0.4	0.0–2.3
Attention deficit hyperactivity disorder	2	0.8	0.1–3.0
Autism	1	0.4	0.0–2.3
Bipolar disorder	20	8.3	5.1–12.5
Depression	10	4.1	2.0–7.5
Epilepsy	8	3.3	1.4–6.4
Other seizure disorders	34	14.1	9.9–19.1
Post-traumatic stress disorder	1	0.4	0.0–2.3
Psychosis	31	12.8	8.9–17.7
Schizoaffective disorder	15	6.2	3.5–10.0
Schizophrenia	114	47.1	40.7–53.6
Somatic symptom disorder	2	0.8	0.1–3.0
Suicidal ideation	1	0.4	0.0–2.3
Other significant emotional or medically unexplained complaints	2	0.8	0.1–3.0

Data on suicide in the KHDSS was available between the years 2008–2016. One hundred and four people committed suicide between the years 2008 and 2016 with 6 committing suicide in the year 2014, the year for which outpatient estimates for other mental conditions have been provided above. The six persons were between the ages of 19 and 66 years with 5 (83.3%) being males. There were no suicide reports among children (<14 years) with only 7 (6.7%) out of the 104 people who committed suicide being under 20 years. This data only included reports which were marked as “intentional self-harm” and so does not include relatively common category of “indeterminate cases” some of which may include suicides. The total number of people who committed suicide in the whole county is unknown, since KHDSS only represents a part of the county. The average number of contacts (an interaction e.g. an intake interview, a treatment session, a follow-up visit involving a user and a staff member on an outpatient basis) per user is unknown.

There is no active community follow-up care for people with mental illnesses, some people report back to the clinics when their psychiatric conditions have deteriorated. In terms of the available treatment, some (21–50%) of the outpatient facilities offer unstructured psychosocial treatments. Sixty-seven percent of mental health outpatient facilities have at least one psychotropic medicine of each therapeutic class (anti-psychotic, antidepressant, mood stabilizer, anxiolytic, and antiepileptic medicines) available in the facility or a near-by pharmacy all year round. There are no day treatment facilities, forensic facilities, community residential facilities, or mental hospitals available in the County.

#### Human rights and equity

The status of voluntary/involuntary admission to general hospitals, which serve as admission facilities for mental health patients, is in general not taken into account. However, it is estimated that the majority of admissions are involuntary. The proportion of patients who were restrained or secluded at least once within the last year in all facilities is unknown. Most violent patients visiting psychiatric outpatient units are chained by caregivers, but it is difficult to precisely know for how long this happens before hospitalisation. However, reports from our community fieldworkers suggest that the patients are isolated and tethered at home for sustained periods.

There are no beds allocated for psychiatry within the County, not even in Kilifi County Hospital, the only referral hospital in the region. This greatly limits access to care as admission is dependent upon availability of beds in the general hospital. There is equity of access to mental health services for other minority users (e.g., linguistic, ethnic, religious minorities) within the County.

### Mental health in primary health care

#### Training in mental health care for primary care staff

The proportion of primary health care doctors and nurses who have received at least two days of training in mental health is unknown. Both physician based primary health care (PHC) and non-physician based PHC clinics are present in the County. Neither the physician based PHC nor non-physician based PHC clinics have assessment and treatment protocols for key mental health conditions. None of the physician-based PHC clinics makes at least one monthly referral to a mental health professional. This is also true for non-physician-based PHC clinics. However, there are two non-governmental clinics for people with epilepsy in the County. One is a research based clinic for care of people with epilepsy and neurodevelopmental disorders runs twice a week under the auspice of Kilifi County Hospital and KEMRI-Wellcome Research Programme and the other is managed by the Foundation for People with Epilepsy, a non-governmental organization, and is run on weekdays. This former clinic focuses on follow-up care for research participants of neurological and mental health studies and does not have the capacity to offer services to all residents of Kilifi County.

As for professional interaction between primary health care staff and other care providers, all or almost all (81–100%) primary health care doctors interacted with a mental health professional at least monthly in the last year often in informal meetings. None of physician-based PHC facilities, non-physician-based PHC clinics, or mental health facilities interacted with a complementary/alternative/traditional practitioner. Traditional healers are first point of care for people with mental health persons in this county and have expressed willingness to refer mental health patients to biomedical facilities if proper referral frameworks are put in place (unpublished work).

#### Prescription in primary health care

Nurses and non-doctor/non nurse primary health care workers are not allowed to prescribe psychotropic medications in any circumstance. However, primary health care doctors are allowed to prescribe psychotropic medications without restrictions. As for availability of psychotropic medicines, few physician-based PHC clinics and non-physician based PHC clinics have at least one psychotropic medicine of each therapeutic category (anti-psychotic, antidepressant, mood stabilizer, anxiolytic, and antiepileptic). The records of the drugs are usually not up-to date and erratic supply of these drugs is common.

### Human resources

#### Human resources in mental health care

The total number of health care professionals working in public mental health facilities is 11.1 per 100,000 population. The total number of professionals in private facilities is unknown. The only personnel available at the mental health outpatient facilities are mental health nurses, social workers and occupational therapists. There are no psychiatrists, psychologists or other mental health workers. The breakdown according of professions per 100,000 population is summarized in Table 2.

**Table 2:** Human resource per 100,000 population

Human resource	Number per 100,000 population
Psychiatrists	0.0
Nurses	0.2
Psychologists	0.0
Social workers	0.2
Occupational therapists and other health or mental health workers^a^	0.4

^a^This includes auxiliary staff, non-doctor/non-physician primary health care workers, health assistants, medical assistants, professional and paraprofessional psychosocial counsellors

Figures provided are best estimates based on official registration and data from professional associations. All the estimates provided refer to human resource in the outpatient mental health facilities, which are the only mental health facilities in the County.

#### Training professionals in mental health

The number of professional graduates from Kilifi County in the last academic year is unknown. No mental health care staff in Kilifi County attended refresher training on the rational use of psychotropic drugs, psychosocial interventions, or child/adolescent mental health issues. Documentation of these numbers is not a priority of the County department of health.

#### Consumer and family associations

There are no consumer associations or family associations in Kilifi County. The government does not provide economic support for either consumer or family associations. Only one non-governmental organization called Afya Research Africa is formally known to be involved in individual assistance activities such as counselling, housing, or support groups.

### Public education and links with other sectors

Data on public education and links with other sectors could not be collected using the brief version of the WHO-AIMs assessment instrument.

#### Monitoring and research

No formally defined list of individual data items that ought to be collected by all mental health facilities exists. The government health department received data from all the three mental health outpatient facilities. However, no report was produced using the data transmitted to the government health department. The data were not detailed and included a few items as follows: all three facilities provided data on the number of users treated and the diagnosis of each patient, two of the three facilities provided data on the number of users restrained and no facility provided data on the user contacts in the facility. Research in Kilifi County is focused on epidemiological surveys of the burden of mental disorders often conducted by researchers from KEMRI-Wellcome Trust Programme. However so far, these studies have only been based on a few mental health problems such as behavioural disorders in young children and epilepsy in all ages [10, 11]. The research consists of monographs, theses and publications in indexed journals.

## Discussion

The findings of this study reveal a fragile mental health system in Kilifi County. Most conspicuously is the lack of mental health care facilities (e.g. no admission beds for mental health patients in the county referral hospital) and the limited human resource allocated to mental health care (e.g. low physician/patient ratio and lack of qualified psychiatrists). There are no legislation and policy frameworks on mental health care in the county and rights of people with mental illnesses are violated (e.g. being restrained). The frequency of psychiatric conditions treated at the outpatient facilities shows that conditions such as depression may be under-recognised in this community as in other low income settings [12, 13]. This study also sheds light on the challenges posed by a devolved system of health care and outdated legislation.

### Psychiatric conditions admitted to hospital

Verbal reports from the nurses managing psychiatric facilities in Kilifi indicate that over 2000 people were diagnosed with a psychiatric condition in 2014, translating to 180 per 100,000 population (95% CI 155–204). This data is not reliable and may be is a gross underestimate of the true burden of mental health conditions in the community as it likely represents severe patients, who access the hospital, and those without animistic beliefs about biomedical treatment. Psychosis and seizures were the commonest diagnosis, which is unsurprising since the long-standing studies on epilepsy and other seizure disorders in this area may have sensitised the community [10], while psychosis has overt symptoms easily identifiable for referral for treatment. The few cases of depression may suggest that this condition is under-recognised in the community due to its internalising symptoms or is considered less debilitating than psychosis or epilepsy, calling for the need to train primary health to identify the condition or to conduct awareness and epidemiological studies to sensitise the community. Suicide within the KHDSS was documented as “intentional self-harm”. There reports of suicide from verbal autopsies within the KHDSS were fewer than those of “indeterminate cases” [14]. This could be due to under-reporting of suicide cases, probably because of the stigma and isolation that family members of people who die by suicide face in many African communities. Indeed a study by Kizza et al. [15] from Uganda indicated that suicidality was associated with “loss of masculinity” and such stereotypes may interfere with reporting of suicide cases, leading to gross under-estimation of these cases. Also, the preponderance of male suicides in our report is consistent with other studies [15, 16]. Further studies are required to understand the reasons for males killing themselves more than females. Suicide cases may be a reflection of the devastating effects of unaddressed depression among the casualties. Few children presented to outpatients clinics with neurodevelopmental and behavioural/emotional problems, probably because most parents cannot identify symptoms for these conditions in their children and they have a misperception that their children will outgrow the disorders. While the numbers provided in this study only represents a proportion of the true burden in the community, they are expected to exert an enormous burden on the County Health System [17], which appeared ill-equipped to address mental health problems as discussed in subsequent sections below.

### Policy and legislative framework

Kenya promulgated a new constitution in 2010, but Mental Health Acts were given little attention in the new dispensation. In fact, the Mental Health Act was last revised in 1989. This act is outdated and does not address the current needs of people with mental illnesses and the unprecedented challenges of a devolved system of health care, which was inaugurated with the new constitution. For instance, the District Mental Health Board, which is supposed to be the mental health authority at the lowest level of administration, is not functional in Kilifi County and there is no County Mental Health Authority [18]. Also, there is no mental hospital in Kilifi County despite the substantial burden of people with mental and neurological disorders [19]. Because of lack of appropriate legislations and policy frameworks, the rights of people with mental illnesses are violated (e.g. being restrained and isolated), little resources are allocated to mental health care (e.g. no budget of staff training) and there is lack of will to use available research findings to change policies on mental health care. Fortunately, there were proposed amendments to improve the Mental Health Act in 2014, but this bill is yet to become law. This is indicative of lack of effective advocacy for mental health in parliament. There has been a positive step towards improving mental health policy with the launch of Kenya’s first mental health policy in May 2016. This was however not captured in our study as data was collected in 2015. Although the new policy brings with it a lot of promise for improved mental health care, the challenge lies in implementing it, especially in remote resource limited settings such as Kilifi.

### Mental health services resources and financing

Data collected with the brief version of the WHO-AIMS suggests that the current budgeting system is not program based; making it difficult to directly determine the exact amount of funds allocated to mental health services. Although the County budget is a publicly accessible document, the implementation process is not, making accountability for resources spent on mental health care difficult, unlike in other countries such as Uganda, where budgetary allocation to mental health is quantified (approximately 4%) [20]. It is possible that the lack of quantification of exact mental health needs in the county makes planning for mental health services difficult. We hope that our efforts to conduct this situation analysis will identify exact mental health needs in the county that may inform objective financial allocation on mental health care.

There is no mental hospital or mental health facility allocated to child or adolescent mental health care despite the substantial burden of mental and neurological disorders particularly in older children [21]. This lack of resources could be due to a number of reasons: (1) until recently, Kilifi as an administrative unit was dependent on Mombasa county (which is located 60 km away and was the provincial headquarters) for essential mental health services; (2) there has been no data on the prevalence of mental, neurological and substance used disorders for informing policy and resource allocation; (3) most people with mental, neurological and substance used disorders visit traditional healers as their first point of care due to misconceptions, stigma and discrimination associated with mental illnesses and therefore the enormous burden of mental, neurological and substance used disorders is unappreciated by biomedical facilities (unpublished work); and (4) meagre financial budget towards mental health care and training of health staff at higher institutions of learning. It is worth emphasising that these reasons alone cannot fully justify the conspicuous lack of resource allocation towards mental health, given that some reasonable resources are spent on other health problems particularly control of infectious diseases such as malaria and HIV, and Expanded Programmes on Immunizations.

Due to the under-resourced mental health care, Kilifi County relies on two psychiatric nurses who serve at the only two public psychiatric outpatient facilities. The low staff patient ratio for mental health poses a risk of poor quality of mental health services, especially diagnosis and management of common mental illnesses. Because of the enormous burden on the health care system, it is likely that only severe psychiatric cases will visit the outpatient clinics, perhaps explaining the few cases of common mental, neurological and substance used disorders reported in the County. For instance only 4% of patients who seek care in these facilities are diagnosed with depression, which may be misperceived as a less severe condition than psychosis; depression contributes to the largest disability adjusted life years for all illnesses in Kenya (2.3%) and worldwide [22].

### Human rights and equity

Stigma and discrimination towards people with mental illness is ubiquitous [23]. Although WHO-AIMS could not assess the extent of human rights violations, it helped highlight a notable absence of bodies that oversee human rights of persons with mental illnesses in facilities. This situation is not unique for Kilifi and can be compared to Uganda [20] although human rights review bodies exist in some sub-Saharan countries like Ghana [24]. Future studies are required to systematically document the extent of discrimination and human rights violations among mental health patients in this area, using standardised methods.

### Mental health in primary health care

Kilifi County and indeed Kenya in general is still lagging behind as far as integrating mental health into primary health care is concerned compared to other countries in sub-Saharan Africa [20, 24]. In Kilfi, care is still offered in outpatient facilities only. Anecdotal evidence suggests that most primary health care providers are willing to be involved in providing mental health care but they require refresher training and guidance to be able to offer this care adequately. Introducing a mental health training intervention for primary health care workers may be a useful way of improving this situation.

### Limitations

There are a number of limitations of this study. The principal investigator in this pilot study (MB) had spent very little time with the respondents prior to collecting this data and may not have built enough trust to get accurate responses from the respondents. Client records for mental health services were scanty and poorly recorded which made the verification of quantitative data virtually impossible. The possible areas of action suggested by the authors were based solely on the evidence generated by this study with no consultation between the authors and the stakeholders regarding their views on priority areas for action. This limits the extent to which these recommendations are acceptable and replicable.

### Possible areas for action

Based on the findings of this study, we provide recommendations using two criteria. We consider the evidence base from this study, other studies within the country and the opportunities provided by the current devolved system of government and of healthcare. These actions rely on cooperation and collaboration between Kilifi, neighbouring counties and with the national government, and are informed by results of studies done within the country, as referenced in each section.

### Short term areas for action

#### Service development priorities

Primary health care workers should receive refresher training on mental health. Some topics for this training should include identification and management of depression, psychosis, suicide, child and adolescent issues, alcohol abuse, anxiety disorders and patients with chronic complaints. Jenkins and colleagues in their study on the experiences and perspectives of trained primary health care workers in Nyanza Kenya, found that even in the presence of a weak health system and inadequate medicine supply, providing mental health training had a positive impact on the quality of life of their clients [25]. However, as shown in a randomized control trial of a mental health training intervention of primary health workers in three districts in Kenya, short term training improved patients’ outcomes, but not ability to diagnose mental illnesses [26]. As such, refresher training should perhaps be done routinely if any long-term benefits are to be realised. Fortunately, these training initiatives could be sustained through the existing training programmes run by the Kenyan ministry of health, which trains health workers from rural health centres. This program uses curriculum developed through a partnership with WHO. The training covers core concepts of mental health, common mental and neurological disorders and health system issues including policies and legislations, but it is yet to be rolled out to the county hospitals in the current devolved system of governance. An evaluation of the impact of the first 1000 trainers showed a 35% mean improvement in knowledge among those who were trained [27], yet the training initiative has not been applied in many rural county and sub-county hospitals in Kenya. Health stakeholders of a region initiate the process of training and as such, the County chief officer of health in Kilifi should initiate this process.

The County government should also initiate talks with traditional healers, who are involved in the diagnosis and treatment of patients with mental health problems [28], to encourage them to refer people with mental health problems to health facilities. Traditional healers are first point of contact for people with mental illnesses, and they have expressed willingness to cooperate if such an initiative was put in place.

#### Advocacy and awareness campaigns

The County government together with stakeholders in mental health should cooperate in initiating and sustaining routine awareness campaigns to create knowledge of the causes and symptoms of common mental health problems. Currently, the national government and non-governmental organizations organize such campaigns. They are held in major cities only on days relevant to mental health such as The World Mental Health Day and International Epilepsy day. A starting point should be conducting these campaigns at the county level and encouraging consumers of mental health services and their families to share their experiences with the community during such campaigns. These campaigns should encourage help seeking and better coping strategies for people with mental health problems. In future, these advocacy and awareness campaigns should also target the communities where people with mental illnesses live by supporting and strengthening existing self-help groups [29].

#### Policy and legislation priorities

Although there was no mental health policy during the time of data collection for this study, a new national policy is now in place. The County Department of Health should organize workshops to sensitize health care providers on the contents of this policy and to encourage them to be part of the implementation process. If implemented, this policy will improve the quality of services provided to people with mental health problems.

### Medium/long term areas for action

#### Research priorities

Epidemiological studies on the spectrum of common mental disorders should be conducted on persons of all age groups to provide reliable data on the burden of mental, neurological and substance use disorders in Kilifi. The results of such studies will be useful in two ways. Firstly, people identified with these mental disorders will receive follow up care through the ministries of health in collaboration with the research centres involved. Secondly, stakeholders can use reliable data generated from these studies to support advocacy for increased budgetary allocation towards mental health services. Additionally, as with any epidemiological studies, there may be increased community awareness because of the research activities. This should however be complemented with active and consistent awareness campaigns.

#### Service development priorities

There should be integration of mental health care into primary care, a model which has been shown to be effective in Kenya [30] and other resource poor settings [31]. This can be done by providing training on WHO’s mental health Gap Action Program Intervention Guide (mhGAP-IG). However, contextualizing the available evidence is paramount.

There is need to increase capacity for managing mental health problems. This requires both an increase in infrastructure and in human resource. Kilifi can utilise the National Health Sector Human Resource Strategy 2014–2018 [32] which clearly outlines the implementation process for increasing human resource in health in every County. It describes the recruitment targets per annum in general and specialized areas including mental health. It also outlines the resources required to train these personnel and the sources of funding for counties to implement this strategy. It also lays emphasis on the importance of cooperation between Counties. Kilifi County has two facilities currently training medical personnel and many more in the neighbouring Mombasa County. Mental health facilities in Mombasa have qualified psychiatric staff, capable of providing training in the teaching facilities in Kilifi. It is therefore feasible to create a coordinated framework where mental health training can be offered in Kilifi by personnel from Mombasa and other neighbouring counties. There is also need to explore if telehealth could be used to offer training to health workers in Kilifi by psychiatrists from Nairobi. A systematic review on the use of information communication technology (ICT) in health initiatives in Kenya found that of the 14 marginalized counties in Kenya [33], Kilifi had the highest number of initiatives [34]. Although none of these initiatives were specifically for mental health, this review indicates that Kilifi has the capacity to utilize ICT in health.

Investment in mobile outpatient assistance teams will help cover distant areas and improve access to services hence avoid hospitalization. Mobile teams could also backup local primary health care services assisting mental patients. Mental health clinics and wards in health facilities should also be established. These will decongest the outpatient facilities.

Lastly, providing community care for patients (and their families) will avoid the costs of hospitalization and promote integration, as well as reduce stigma associated with institutionalized care.

#### Advocacy and awareness campaigns

Stakeholders in mental health care i.e. consumers and families should be encouraged to form associations through which they can be involved in processes aimed at improving care.

#### Policy and legislation priorities

Legislation should be updated to protect the rights of people with mental health problems such as equitable access to care, protection from inhuman treatment and involuntary admissions. This requires more consistent advocacy efforts from all mental health stakeholders. Advocates of people with mental health problems should work together towards the same goal. Indeed lack of cooperation among stakeholders has been an obstacle in the efforts to improve mental health services in resource poor settings [35]. Campaigns such as those started by the National Department of Mental Health services to decriminalize suicide should be made into laws, to protect people with suicidal ideations and encourage them to seek help. Currently, mental health governance is still centralized. This limits the extent to which counties can independently plan and implement mental health activities. Devolution of mental health services should be hastened to enable each county respond to the specific mental health needs of their regions.

## Conclusions

Mental health care system in Kilifi Kenya is under resourced and should be improved through several approaches and strategies. There is an urgent need to increase resources allocated for mental health. Infrastructure and human resource should be increased. Policy and legislations need to be put in place to protect the rights of people with mental illnesses. Mental health should be integrated with a strengthened primary care to increase access to services.
